# Supplementary material for: Diagnostic and Prognostic Characteristics of Circulating Free DNA Methylation Detected by the Electrochemical Method in Malignant Tumors
Source: Cancers (Basel). 2021 Feb 7;13(4):664. doi: 10.3390/cancers13040664 (PMC7914975; doi:10.3390/cancers13040664)
Supplement: Supplementary file 1 [file cancers-13-00664-s001.pdf]

Supplementary Materials: Diagnostic and Prognostic Characteristics of Circulating Free DNA Methylation Detected by the Electrochemical Method in Malignant Tumors

Li-Yue Sun, Zi-Ming Du, Yu-Ying Liu, Yan-Hong Li, Xiao-Min Liu, Ting Wang and Jian-Yong Shao

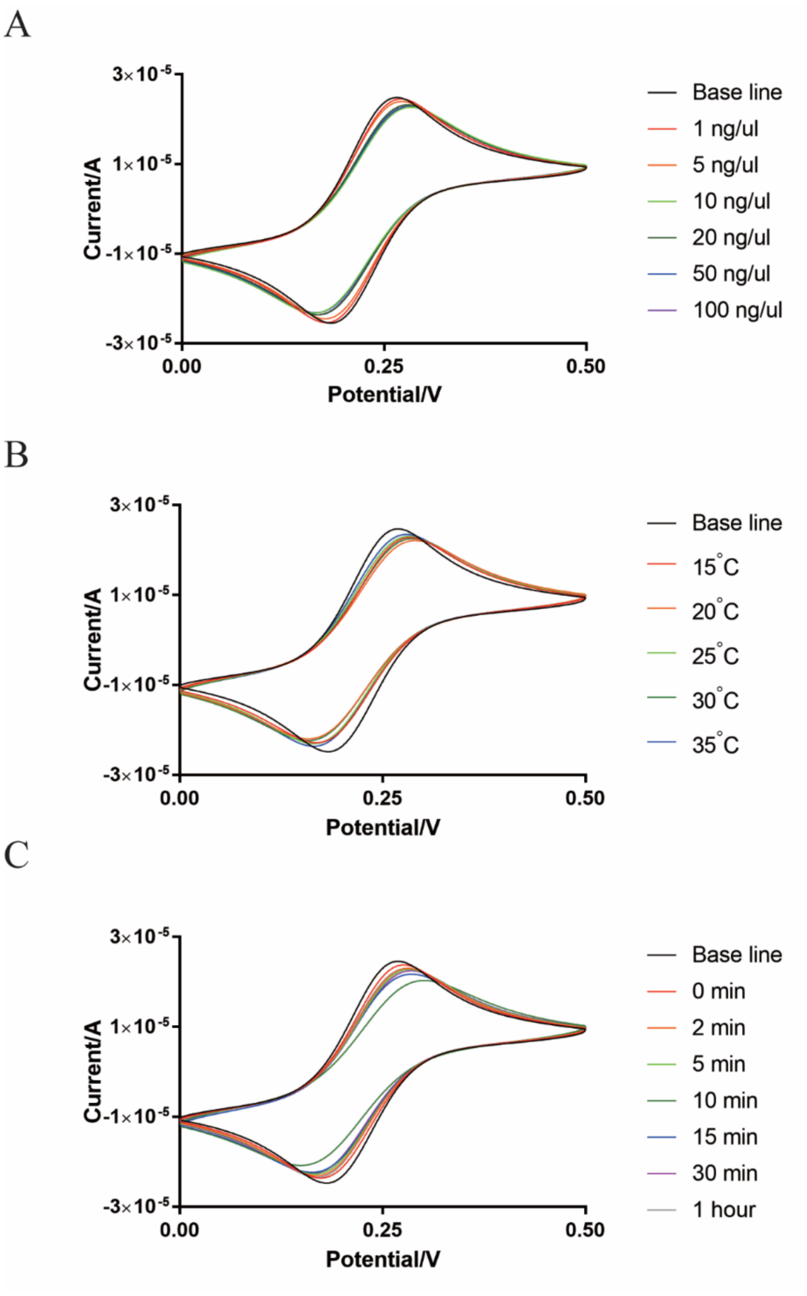

Figure S1. (**A**). Change of CV curve after adsorption of different concentrations of DNA. The results showed that the CV curve changed the most when the DNA concentration was 10 ng/uL. (**B**). Changes in CV curve after DNA adsorption at different temperatures. The results showed that the CV curve changed the most when the ambient temperature is 20 °C. (**C**). Changes in CV curve after DNA adsorption for different time range. The results showed that the CV curve changed the most when the DNA adsorption time was 10 minutes.

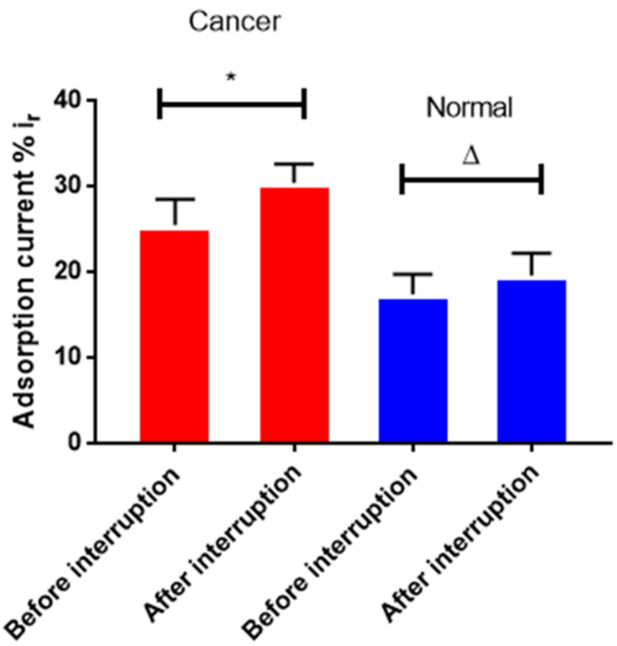

Figure S2. After ultrasound interrupted the DNA of tumor and normal tissue, it was found that tumor tissue DNA after interruption was higher than that before interruption (after interruption vs. before interruption, 30.37 ± 2.17% vs. 25.44 ± 3.06%, $p = 0.019$). There was no significant difference between normal tissue DNA after interrupted and before interrupted (after interruption vs. before interruption, 19.53 ± 2.64% vs. 17.36 ± 2.36%, $p = 0.207$). Δ $p > 0.05$, * $p < 0.05$.

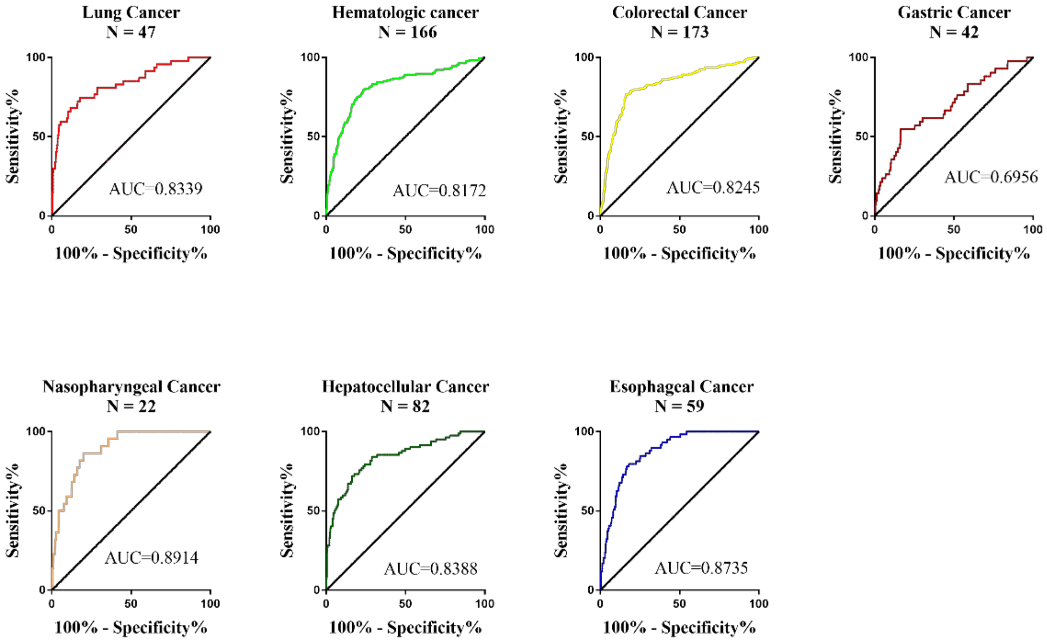

Figure S3. ROC curve of electrochemical detection of cfDNA adsorption rate in the diagnosis of 7 kinds of malignant tumors. In 47 case of lung cancer, the sensitivity was 74.46% (35/47) and AUC of ROC curve was 0.8339. In 166 case of hematological cancer, the sensitivity was 74.70% (124/166) and AUC of ROC curve was 0.8172. In 173 case of colorectal cancer, the sensitivity was 78.61% (136/173) and AUC of ROC curve was 0.8245. In 42 case of gastric cancer, the sensitivity was 54.76% (23/42) and AUC of ROC curve was 0.6956. In 22 case of nasopharyngeal cancer, the sensitivity was 81.82% (18/22) and AUC of ROC curve was 0.8914. In 82 case of hepatocellular cancer, the sensitivity was 73.17% (60/82) and AUC of ROC curve was 0.8388. In 59 case of esophageal cancer, the sensitivity was 79.66% (47/59) and AUC of ROC curve was 0.8735.

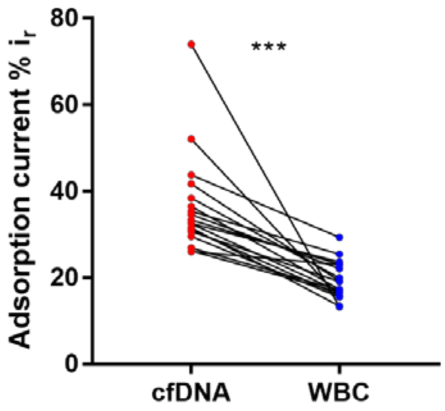

Figure S4. The line diagram showed the cfDNA adsorption rate and peripheral white blood cell (WBC) DNA adsorption rate of 18 patients with malignant tumor. The results showed that the adsorption rate of cfDNA was higher than that of WBC DNA in the same patient.

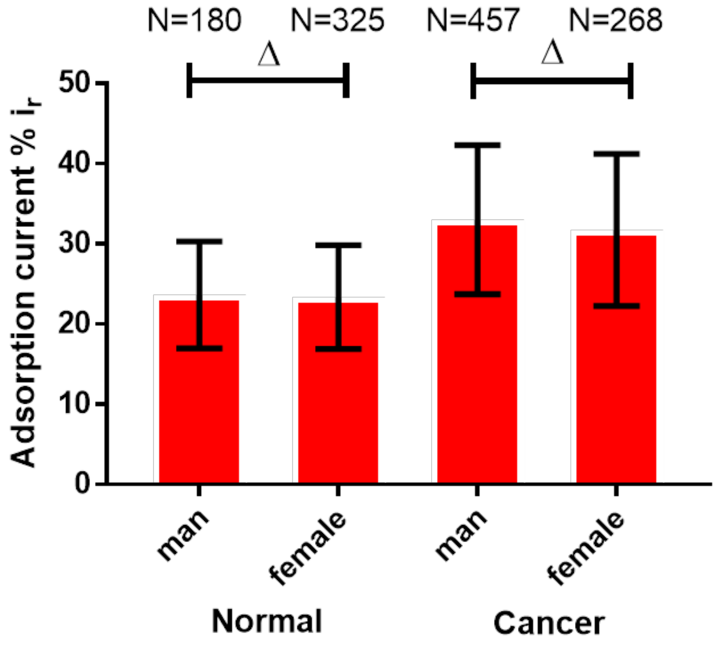

Figure S5. Histogram of cfDNA adsorption rate of normal people and initially diagnosed patients with cancer of different genders. The results showed that there is no difference in the cfDNA adsorption rate between normal people (male vs. female, 23.19 ± 6.26% vs. 24.43 ± 7.36%)and malignant tumor patients (male vs. female, 33.02 ± 9.31% vs. 24.43 ± 7.36%) of different genders. Δ $p > 0.05$.

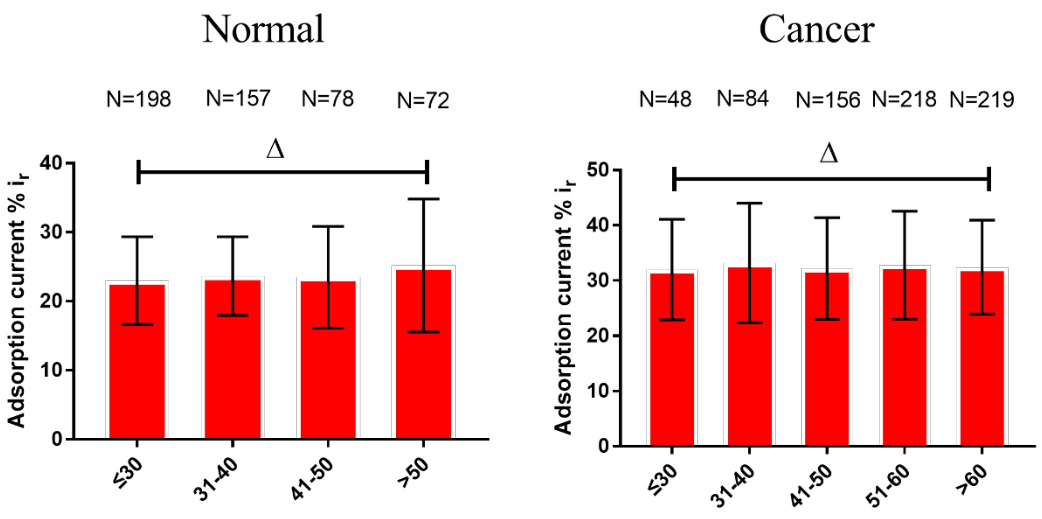

Figure S6. Histogram of cfDNA adsorption rate of normal people and initially diagnosed patients with cancer of different ages. In the normal people, the results showed that cfDNA adsorption rates of ≤30 age, 31–40 age, 41–50 age, and >50 age were 23.00 ± 6.36%, 23.66 ± 5.70%, 23.48 ± 7.36% and 25.19 ± 9.63%, respectively. In the cancer patients, the results showed that cfDNA adsorption rates of ≤30 age, 31–40 age, 41–50 age, 51–60 age, and >60 age were 32.00 ± 9.10%, 33.20 ± 10.84%, 32.18 ± 9.21%, 32.78 ± 9.78% and 32.43 ± 8.49%, respectively. The results showed that there is no difference in the cfDNA adsorption rate between normal people and malignant tumor patients of different ages. Δ $p > 0.05$.

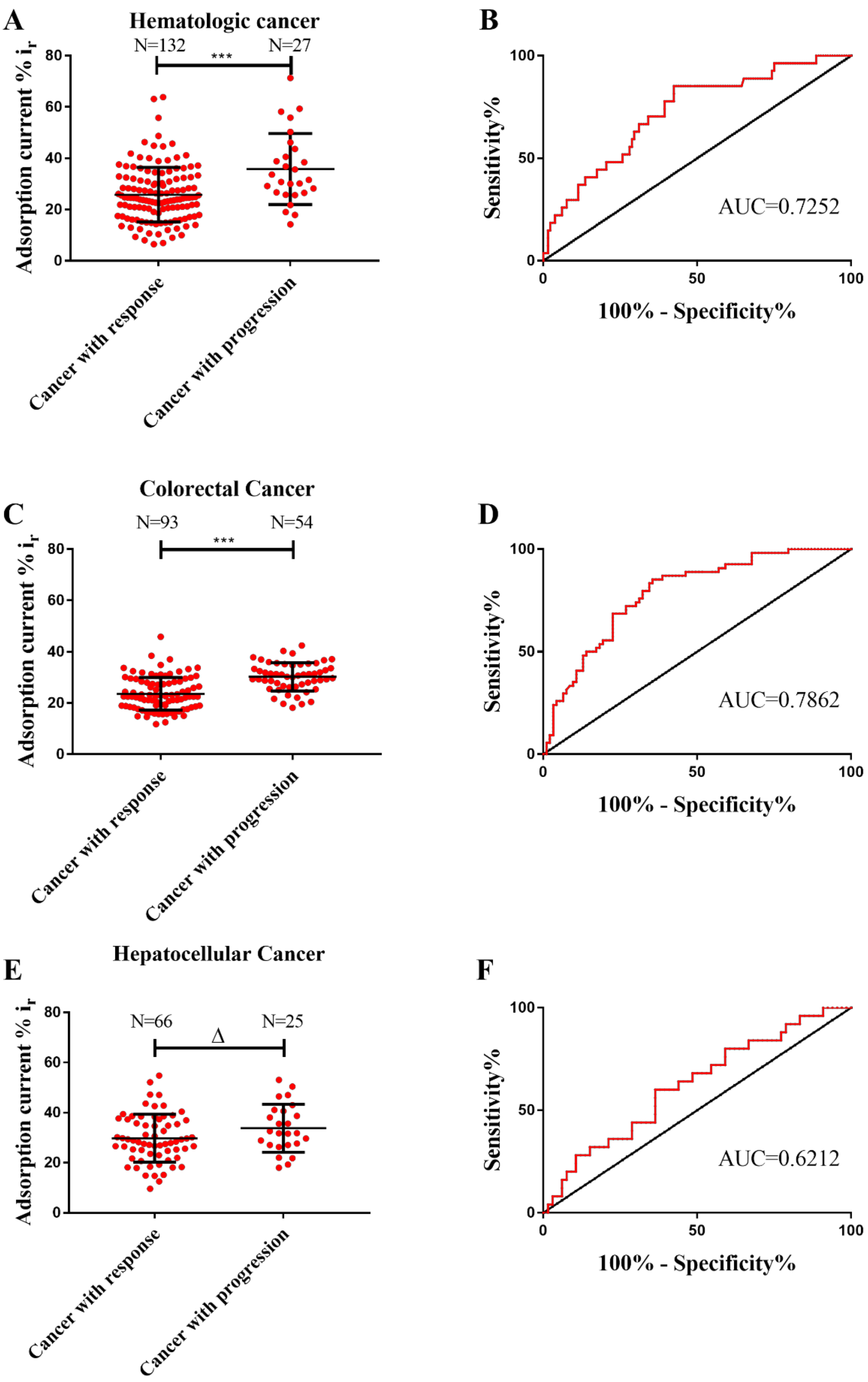

Figure S7. (**A**). Among hematological cancer, a total of 132 patients with response after treatment and 27 patients with recurrence/progress after treatment were tested. The results showed that the cfDNA adsorption rate of patients with response was 25.80 ± 10.63% and the cfDNA adsorption rate of patients with recurrence/progress was 35.78 ± 13.84%. The cfDNA adsorption rate of patients in the recurrence/progress group was significantly higher than patients with response. (**B**). The ROC curve shows that the AUC of ROC curve was 0.7252, the sensitivity of cfDNA adsorption rate in monitoring

the curative effect of hematological cancer is 66.67% and the specificity is 68.18%. (**C**). We tested a total of 93 colorectal cancer patients with response after treatment and 54 patients with recurrence/progress after treatment. The results showed that the cfDNA adsorption rate of patients in response was 23.60 ± 6.39%, the cfDNA adsorption rate of patients with relapsed/progressed was 30.22 ± 5.49%, and the cfDNA adsorption rate of patients who relapsed/progressed after treatment was significantly higher. (**D**). The ROC curve shows that the AUC was 0.7862, the sensitivity of cfDNA adsorption rate in monitoring the efficacy of colorectal cancer is 68.52%, and the specificity is 76.34%. (**E**). We tested 66 cases of hepatocellular cancer with response after treatment and 25 cases of hepatocellular cancer with relapsed/progressed after treatment. The results showed that the cfDNA adsorption rate of response patient treatment was 29.79 ± 9.55% and the cfDNA adsorption rate of relapsed/progressed patients was 33.83 ± 9.57%, and the cfDNA adsorption rate of relapsed/progressed patients was higher than patients with response. However, the difference is not statistically significant. (**F**). The ROC curve shows that the AUC is 0.6212. The sensitivity of cfDNA adsorption rate in monitoring the therapeutic effect of hepatocellular cancer is 68.00%, and the specificity is 50.00%. *** $p < 0.001$, Δ $p > 0.05$.

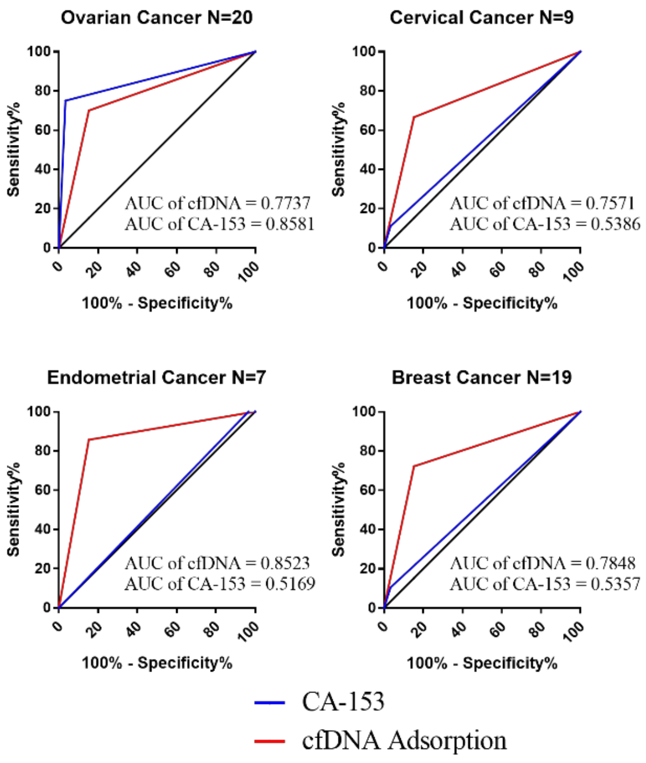

Figure S8. ROC curve of electrochemical detection of cfDNA adsorption rate compared with serum CA-153 level in the diagnosis of 4 malignant tumors. A total of 55 patients with malignant tumors had serum CA-153 levels detected at the time of initial diagnosis, including 9 cases of cervical cancer, 20 cases of ovarian cancer, 7 cases of endometrial cancer, and 19 cases of breast cancer. In addition, a total of 59 cases of normal people received CA-153 level testing. The normal range of serum CA-153 level in this research unit is 0–25 U/mL. The sensitivity of CA-153 detection in these patients was 32.73% (18/55), the specificity was 96.61% (57/59), and the area under the ROC curve was 0.6467. In the same group of patients, the sensitivity of cfDNA adsorption rate was 70.91% (39/55), specificity was 84.75% (50/59), and the area under the ROC curve was 0.7783. The electrochemical method for detecting cfDNA adsorption rate is superior or not inferior to the diagnostic effect of serum CA-153 level in most of malignant tumors. In 20 ovarian cancer patients, the sensitivity of CA-153 and cfDNA adsorption rate was 75.00% (15/20) and 70.00% (14/20), respectively. And the AUC of ROC curve was 0.6889 and 0.7938, respectively. In 9 cervical cancer patients, the sensitivity of CA-153 and cfDNA adsorption rate was 11.11% (1/9) and 66.67% (6/9), respectively. And the AUC of ROC curve was 0.5386 and 0.7571, respectively. In 7 endometrial cancer patients, the sensitivity of CA-153 and cfDNA adsorption rate was 0.00% (0/7) and 85.71% (6/7), respectively. And the AUC of ROC curve was 0.5169 and 0.8523, respectively. In 19 breast cancer patients, the sensitivity of CA-153 and cfDNA adsorption rate was 10.53% (2/19) and 73.68% (14/19), respectively. And the AUC of ROC curve was 0.5357 and 0.7848, respectively.

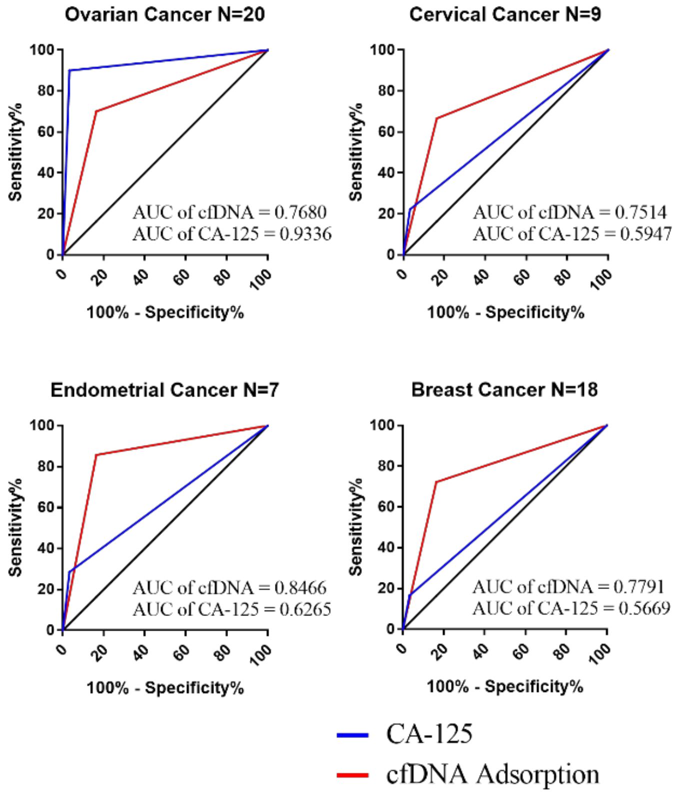

Figure S9. ROC curve of electrochemical detection of cfDNA adsorption rate compared with serum CA-125 level in the diagnosis of 4 malignant tumors. A total of 54 patients with malignant tumors had serum CA-125 levels detected at the time of initial diagnosis, including 9 cases of cervical cancer, 20 cases of ovarian cancer, 7 cases of endometrial cancer, and 18 cases of breast cancer. In addition, a total of 61 cases of normal people received CA-125 level testing. The normal range of serum CA-125 level in this research unit is 0-35 U/mL. The electrochemical method for detecting cfDNA adsorption rate is superior or not inferior to the diagnostic effect of serum CA-125 level in most of malignant tumors. In 20 ovarian cancer patients, the sensitivity of CA-125 and cfDNA adsorption rate was 90.00% (18/20) and 70.00% (14/20), respectively. And the AUC of ROC curve was 0.9336 and 0.7680, respectively. In 9 cervical cancer patients, the sensitivity of CA-125 and cfDNA adsorption rate was 22.22% (2/9)and 66.67% (6/9), respectively. And the AUC of ROC curve was 0.5947 and 0.7514, respectively. In 7 endometrial cancer patients, the sensitivity of CA-125 and cfDNA adsorption rate was 28.57%(2/7) and 85.71% (6/7), respectively. And the AUC of ROC curve was 0.6265 and 0.8466, respectively. In 19 breast cancer patients, the sensitivity of CA-125 and cfDNA adsorption rate was 16.67% (3/18)and 72.22% (13/18), respectively. And the AUC of ROC curve was 0.5669 and 0.7791, respectively.

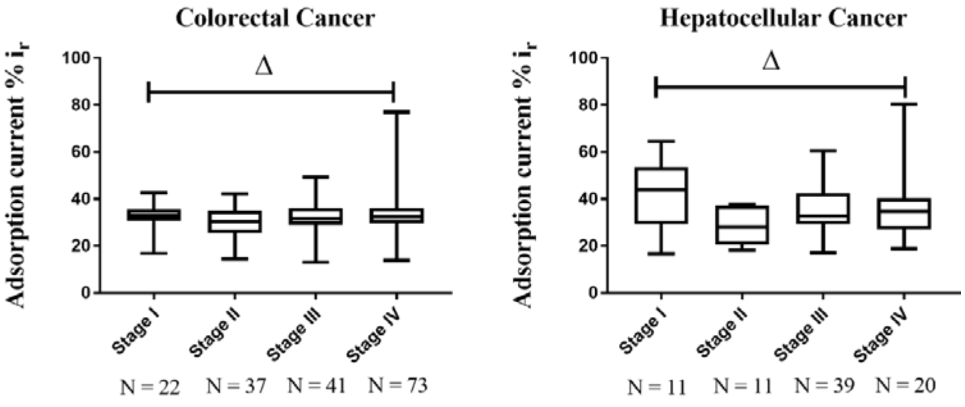

Figure S10. The box plot showed the cfDNA adsorption rate of patients with colorectal cancer and hepatocellular carcinoma in different TNM stages. The results showed that there was no significant difference in the cfDNA adsorption rate between patients with colorectal cancer and hepatocellular cancer with different TNM stages.

Table S1. Clinical characteristics of 20 cases of colorectal cancer, hepatocellular carcinoma tissue.

Characteristics	CRC (*N* = 20)	HCC (*N* = 20)
Age (year)	57(48, 64)	54(47, 63)
Gender, male	15 (75.00%)	16 (80.00%)
Pathology grade		
Good differentiated	9(45.00%)	12(60.00%)
Poorly differentiated	11(55.00%)	8(40.00%)
TNM stage		
I	4(20.00%)	7(35.00%)
II	2(10.00%)	1(5.00%)
III	6(30.00%)	9(45.00%)
IV	8(40.00%)	3(15.00%)

Abbreviation: CRC, colorectal cancer; HCC, hepatocellular carcinoma

Table S2. Sensitivity and specificity of each cancer in detect plasm cfDNA methylation.

Tumor Type	Sensitivity%	Specificity%
Lung cancer	74.46	80.99
Hematologic tumor	74.70	80.99
Colorectal cancer	78.61	80.99
Prostatic cancer	81.81	80.99
Ovarian cancer	70.00	80.99
Cervical cancer	66.67	80.99
Gastric cancer	54.76	80.99
Endometrial cancer	85.71	80.99
Nasopharynx cancer	81.82	80.99
Hepatocellular carcinoma	73.17	80.99
Esophagus cancer	79.66	80.99
Breast cancer	70.83	80.99
Thyroid cancer	81.25	80.99
Pancreatic cancer	58.33	80.99
Cholangiocellular carcinoma	60.00	80.99

Table S3. Clinical characteristics of plasma samples from patients with lung cancer.

Variable	Lung Cancer (*n* = 47)	Percentage%
Age(year)	56.81 ± 9.77	
Sexual, *n* (%)		
Male	36	76.60
Female	11	23.40
TNM stage, *n* (%)		
I	7	14.89
II	0	0.00
III	7	14.89
IV	31	65.96

Unclearly	2	4.26
Differentiate, *n* (%)		
Well-Moderate	12	25.53
Poor-undifferentiated	26	55.32
Unclearly	9	19.15
Histology, *n* (%)		
Adenocarcinoma	32	68.09
Squamous cell carcinoma	12	25.53
Small cell lung cancer	2	4.26
Lymphoepitheliomas carcinoma	1	2.13

Table S4. Clinical characteristics of plasma samples from patients with hematologic tumors.

**Variable**	**Hematologic Tumors (*n* = 166)**	**Percentage%**
Age(year)	48.75 ± 14.99	
Sexual, *n* (%)		
Male	96	57.83
Female	70	42.17
TNM stage, *n* (%)		
I	24	14.46
II	58	34.94
III	35	21.08
IV	38	22.89
Unclearly	11	6.63
Histology, *n* (%)		
Hodgkin's lymphoma	12	7.23
Non-Hodgkins lymphoma	141	84.94
Leukemia	10	6.02
Other	3	1.81

Table S5. Clinical characteristics of plasma samples from patients with colorectal cancer.

**Variable**	**Colorectal Cancer (*n* = 173)**	**Percentage%**
Age(year)	54.07 ± 12.50	
Sexual, *n* (%)		
Male	109	63.01
Female	64	36.99
TNM stage, *n* (%)		
I	22	12.72
II	37	21.39
III	41	23.70
IV	73	42.20
Differentiate, *n* (%)		
Well-Moderate	121	69.94
Poor-undifferentiated	37	21.39
Unclearly	15	8.67

Table S6. Clinical characteristics of plasma samples from patients with prostatic cancer.

**Variable**	**Prostatic Cancer (*n* = 11)**	**Percentage%**
Age(year)	59.09 ± 9.96	
TNM stage, *n* (%)		
I	3	27.27
II	0	0.00
III	1	9.09

IV	7	63.64
Differentiate, *n* (%)		
Well-Moderate	4	36.36
Poor-undifferentiated	7	63.64

Table S7. Clinical characteristics of plasma samples from patients with ovarian cancer.

**Variable**	**Ovarian Cancer (*n* = 20)**	**Percentage%**
Age(year)	53.10 ± 12.11	
TNM stage, *n* (%)		
I	1	5.00
II	4	20.00
III	4	20.00
IV	11	55.00
Differentiate, *n* (%)		
Well-Moderate	1	5.00
Poor-undifferentiated	18	90.00
Unclearly	1	5.00

Table S8. Clinical characteristics of plasma samples from patients with cervical cancer.

**Variable**	**Cervical Cancer (*n* = 9)**	**Percentage%**
Age(year)	45.56 ± 9.99	
TNM stage, *n* (%)		
I	4	44.44
II	2	22.22
III	2	22.22
IV	1	11.11
Differentiate, *n* (%)		
Well-Moderate	5	55.56
Poor-undifferentiated	4	44.44
Histology, *n* (%)		
Adenocarcinoma	1	11.11
Squamous cell carcinoma	8	88.89

Table S9. Clinical characteristics of plasma samples from patients with gastric cancer.

**Variable**	**Gastric Cancer (*n* = 42)**	**Percentage%**
Age(year)	54.74 ± 13.47	
Sexual, *n* (%)		
Male	24	57.14
Female	18	42.86
TNM stage, *n* (%)		
I	8	19.05
II	6	14.29
III	10	23.81
IV	18	42.86
Differentiate, *n* (%)		
Well-Moderate	5	11.90
Poor-undifferentiated	37	88.10

Table S10. Clinical characteristics of plasma samples from patients with endometrial cancer.

Variable	Endometrial Cancer (*n* = 7)	Percentage%
Age(year)	50.71 ± 5.88	
TNM stage, *n* (%)		
I	6	85.71
II	1	14.29
Differentiate, *n* (%)		
Well-Moderate	6	85.71
Poor-undifferentiated	1	14.29

Table S11. Clinical characteristics of plasma samples from patients with nasopharyngeal cancer.

Variable	Nasopharyngeal Carcinoma (*n* = 22)	Percentage%
Age(year)	50.50 ± 9.95	
Sexual, *n* (%)		
Male	18	81.82
Female	4	18.18
TNM stage, *n* (%)		
II	1	4.55
III	9	40.91
IV	12	54.55
Differentiate, *n* (%)		
Well-Moderate	1	4.55
Poor-undifferentiated	21	95.45

Table S12. Clinical characteristics of plasma samples from patients with hepatocellular cancer.

Variable	Hepatocellular Cancer (*n* = 82)	Percentage%
Age(year)	52.54 ± 12.29	
Sexual, *n* (%)		
Male	73	89.02
Female	9	10.98
TNM stage, *n* (%)		
I	11	13.41
II	11	13.41
III	39	47.56
IV	20	24.39
Unclearly	1	1.22
Differentiate, *n* (%)		
Well-Moderate	15	18.29
Poor-undifferentiated	17	20.73
Unclearly	50	60.98

Table S13. Clinical characteristics of plasma samples from patients with esophageal cancer.

Variable	Esophageal Cancer (*n* = 59)	Percentage%
Age(year)	58.86 ± 8.72	
Sexual, *n* (%)		
Male	55	93.22
Female	4	6.78
TNM stage, *n* (%)		
I	3	5.08
II	14	23.73

III	19	32.20
IV	23	38.98
Differentiate, *n* (%)		
Well-Moderate	28	47.46
Poor-undifferentiated	26	44.07
Unclearly	5	8.47
Histology, *n* (%)		
Adenocarcinoma	2	3.39
Squamous cell carcinoma	53	89.83
Small cell lung cancer	1	1.69
Other	3	5.08

Table S14. Clinical characteristics of plasma samples from patients with breast cancer.

**Variable**	**Breast Cancer (*n* = 24)**	**Percentage%**
Age(year)	46.71 ± 11.49	
TNM stage, *n* (%)		
I	4	16.67
II	5	20.83
III	6	25.00
IV	9	37.50
Differentiate, *n* (%)		
Well-Moderate	13	54.17
Poor-undifferentiated	5	20.83
Unclearly	6	25.00

Table S15. Clinical characteristics of plasma samples from patients with thyroid cancer.

**Variable**	**Thyroid Cancer (*n* = 16)**	**Percentage%**
Age(year)	43.38 ± 15.01	
Sexual, *n* (%)		
Male	7	43.75
Female	9	56.25
TNM stage, *n* (%)		
I	12	75.00
II	1	6.25
III	1	6.25
IV	2	12.50

Table S16. Clinical characteristics of plasma samples from patients with pancreatic cancer.

**Variable**	**Pancreatic Cancer (*n* = 12)**	**Percentage%**
Age(year)	60.08 ± 12.77	
Sexual, *n* (%)		
Male	5	41.67
Female	7	58.33
TNM stage, *n* (%)		
I	0	0.00
II	2	16.67
III	2	16.67
IV	8	66.67
Differentiate, *n* (%)		
Well-Moderate	3	25.00
Poor-undifferentiated	3	25.00
Unclearly	6	50.00

Table S17. Clinical characteristics of plasma samples from patients with cholangiocarcinoma.

**Variable**	**Cholangiocarcinoma (*n* = 10)**	**Percentage%**
Age(year)	56.20 ± 9.39	
Sexual, *n* (%)		
Male	8	80.00
Female	2	20.00
TNM stage, *n* (%)		
I	0	0.00
II	0	0.00
III	1	10.00
IV	9	90.00
Differentiate, *n* (%)		0.00
Well-Moderate	2	20.00
Poor-undifferentiated	4	40.00
Unclearly	4	40.00
